# Atrial fibrillation-specific refinement of the STOP-Bang sleep apnoea screening questionnaire: insights from the Virtual-SAFARI study

**DOI:** 10.1007/s00392-023-02157-9

**Published:** 2023-02-11

**Authors:** Konstanze Betz, Dominique V. M. Verhaert, Monika Gawalko, Astrid N. L. Hermans, Zarina Habibi, Nikki A. H. A. Pluymaekers, Rachel M. J. van der Velden, Marloes Homberg, Suzanne Philippens, Maartje J. M. Hereijgers, Bianca Vorstermans, Sami O. Simons, Dennis W. den Uijl, Sevasti-Maria Chaldoupi, Justin G. L. M. Luermans, Sjoerd W. Westra, Theo Lankveld, Reindert P. van Steenwijk, Bernard Hol, Ulrich Schotten, Kevin Vernooy, Jeroen M. Hendriks, Dominik Linz

**Affiliations:** 1 Department of Cardiology, Cardiovascular Research Institute Maastricht (CARIM), Maastricht University Medical Centre, P. Debyelaan 25, 6229 HX Maastricht, The Netherlands; 2 Department of Internal Medicine, Eifelklinik St. Brigida GmbH & Co. KG, Kammerbruchstraße 8, 52152 Simmerath, Germany; 3 Department of Cardiology, Radboud University Medical Center and Radboud Institute for Health Sciences, Geert Grooteplein Zuid 10, 6525 GA Nijmegen, The Netherlands; 4 1St Department of Cardiology, Doctoral School, Medical University of Warsaw, Żwirki i Wigury 61, 02-091 Warsaw, Poland; 5 Institute of Pharmacology, West German Heart and Vascular Centre, University Duisburg-Essen, Forsthausweg 2, 47057 Duisburg, Germany; 6 Department of Anesthesiology, Maastricht University Medical Center, P. Debyelaan 25, 6229 HX Maastricht, The Netherlands; 7 Department of Respiratory Medicine, Maastricht University Medical Centre, P. Debyelaan 25, 6229 HX Maastricht, The Netherlands; 8 Netherland Sleep Institute, Computerweg 4, 3821 AB Amersfoort, The Netherlands; 9 Centre for Heart Rhythm Disorders, University of Adelaide and Royal Adelaide Hospital, Port Rd, Adelaide, SA 5000 Australia; 10 Caring Futures Institute, College of Nursing and Health Sciences, Flinders University, Sturt Rd, Bedford Park, Adelaide, SA 5042 Australia; 11 Department of Biomedical Sciences, Faculty of Health and Medical Sciences, University of Copenhagen, Nørregade 10, 1165 Copenhagen, Denmark; 12 Maastricht UMC+, Maastricht Heart+Vascular Center, 6202 AZ Maastricht, The Netherlands

**Keywords:** Atrial fibrillation, Sleep-disordered breathing, Sleep apnoea, mHealth, STOP-Bang questionnaire, Ablation

## Abstract

**Background:**

Sleep-disordered breathing (SDB) is prevalent in up to 50% of patients referred for atrial fibrillation (AF) catheter ablation (CA). Currently, it remains unclear how to improve pre-selection for SDB screening in patients with AF.

**Aim:**

We aimed to (1) assess the accuracy of the STOP-Bang screening questionnaire for detection of SDB within an AF population referred for CA; (2) derive a refined, AF-specific SDB score to improve pre-selection.

**Methods:**

Consecutive AF patients referred for CA without a history of SDB and/or SDB screening were included. Patients were digitally referred to the previously implemented Virtual-SAFARI SDB screening and management pathway including a home sleep test. An apnoea–hypopnoea index (AHI) of  ≥ 15 was interpreted as moderate-to-severe SDB. Logistic regression analysis was used to assess characteristics associated with moderate-to-severe SDB to refine pre-selection for SDB screening*.*

**Results:**

Of 206 included patients, 51% were diagnosed with moderate-to-severe SDB. The STOP-Bang questionnaire performed poorly in detecting SDB, with an area under the receiver operating characteristic curve (AUROC) of 0.647 (95% Confidence-Interval (CI) 0.573–0.721). AF-specific refinement resulted in the BOSS-GAP score. Therein, BMI with cut-off point ≥ 27 kg/m^2^ and previous stroke or transient ischaemic attack (TIA) were added, while tiredness and neck circumference were removed. The BOSS-GAP score performed better with an AUROC of 0.738 (95% CI 0.672–0.805) in the overall population.

**Conclusion:**

AF-specific refinement of the STOP-Bang questionnaire moderately improved detection of SDB in AF patients referred for CA. Whether questionnaires bring benefits for pre-selection of SDB compared to structural screening in patients with AF requires further studies.

**Trial registration number:**

ISOLATION was registered NCT04342312, 13-04-2020.

**Graphical Abstract:**

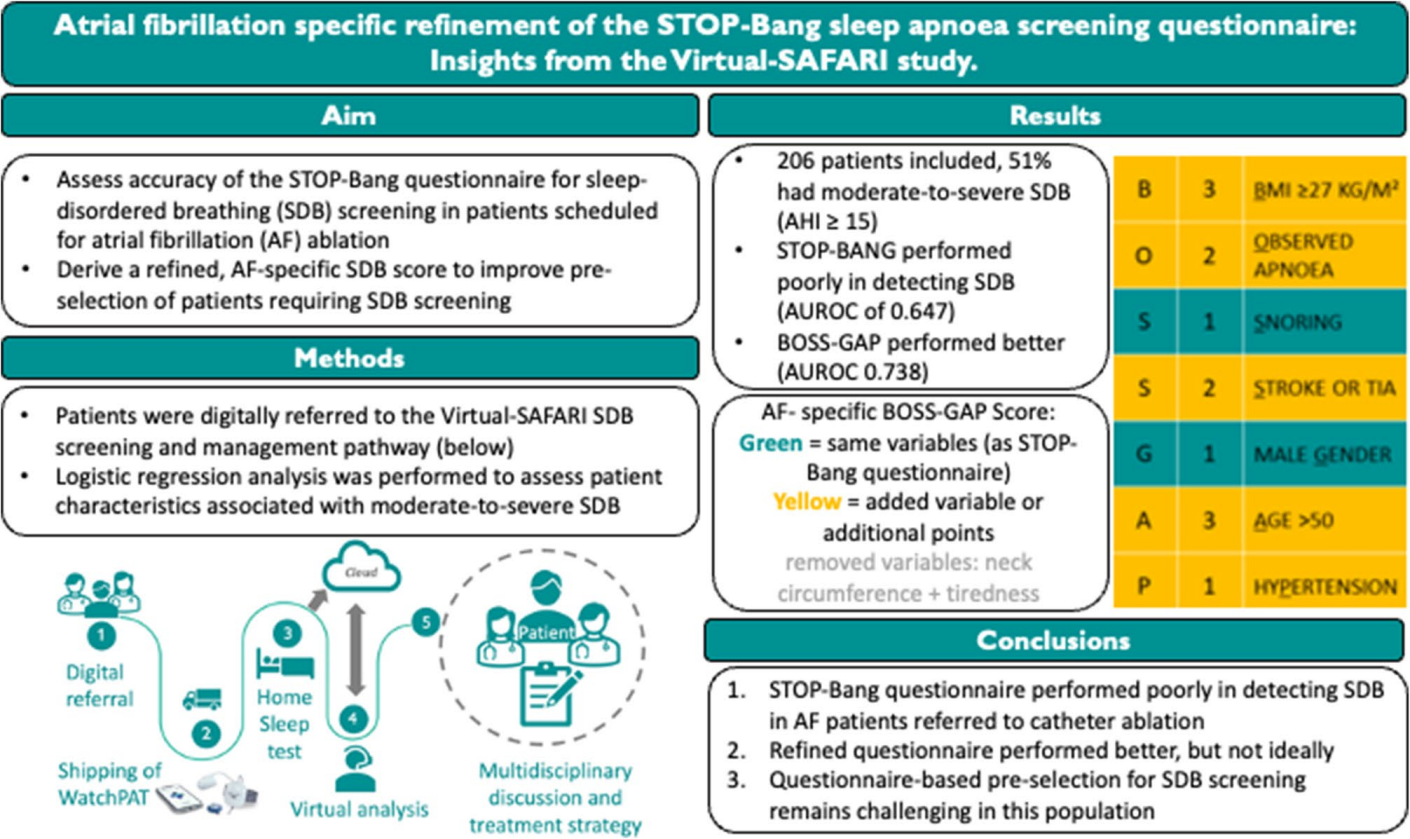

**Supplementary Information:**

The online version contains supplementary material available at 10.1007/s00392-023-02157-9.

## Introduction

Comprehensive risk factor control is one of the main pillars of atrial fibrillation (AF) management. [1] One established risk factor for AF is sleep-disordered breathing (SDB), which is present in up to 50% of all AF patients and is associated with AF progression and increased recurrence rates after AF catheter ablation (CA), when undiagnosed and thus untreated [2, 3].

The limited access to SDB testing complicates the implementation of SDB management in AF patients, as demonstrated in a joint survey by the European Heart Rhythm Association (EHRA) and the European Society of Cardiology’s Association of Cardiovascular Nurses and Allied Professions (ACNAP) [4]. Previously, we introduced the Virtual-SAFARI approach, a remote SDB screening and management pathway using a simple and validated peripheral arterial tone (PAT) based home sleep test in a cohort of consecutive AF patients scheduled for CA. This structural screening approach was feasible, fast, and accompanied by high patient satisfaction [5]. Nevertheless, implementing such a structural screening approach in the workup of patients with AF may lead to an increased burden of healthcare resources and costs. Identifying patients most likely to have SDB might limit this burden. Therefore, the question remains whether the pre-selection process of patients requiring SDB testing can be further optimized to better use the available resources for SDB screening.

Herein, we assessed the accuracy and performance of the STOP-Bang questionnaire, a widely accepted pre-selection tool for SDB screening [2, 6, 7], for detection of SDB in a cohort of patients with AF scheduled for CA who underwent a home sleep test. We further aimed to improve pre-selection of patients requiring SDB screening by an AF-specific adjustment of the STOP-Bang questionnaire.

## Materials and methods

In the Maastricht University Medical Center (MUMC +) and Radboud University Medical Center (Radboudumc), consecutive patients referred for CA undergo systematic screening for common comorbidities and triggers for AF, including SDB. In this context, patients complete the STOP-Bang questionnaire and are subsequently referred to a virtual SDB screening and management pathway, irrespective of results of the questionnaire, unless they have been previously diagnosed with SDB, have recently completed a sleep test that indicated absence of SDB, or refuse SDB screening. A detailed description of this approach is provided elsewhere [5]. In brief, patients are educated about the interaction between AF and SDB and are digitally referred to a virtual sleep lab. Within 1–3 weeks, they receive a WatchPAT-ONE or WatchPAT 300 device at home. After a one-time overnight use of the device, the recordings are submitted to a sleep physician via a secured cloud. The sleep physician reviews the results and discusses the diagnosis with the patient and referring physician.

### Study population

This is a sub-study of the ongoing ISOLATION cohort study (NCT04342312) and ISOLATION ‘light’ registry [8]. These studies prospectively enrol consecutive patients with symptomatic paroxysmal or persistent AF referred for CA in the MUMC + or Radboudumc. The ISOLATION cohort study and ISOLATION ‘light’ registry were approved by the ethical review boards MUMC + /Maastricht University (METC numbers 19-052, 2019-1022) and Radboudumc (METC number 2019-5629) and comply with the Declaration of Helsinki. All participants provided written informed consent.

Patients were eligible for this study if they were included between October 2020 and January 2022. Patients were excluded if they did not undergo the remote sleep test, if they failed to complete the STOP-Bang questionnaire, or if the time between completing the STOP-Bang questionnaire and the sleep test was more than 6 months.

### Sleep apnoea diagnosis

SDB was diagnosed using the WatchPAT-ONE or WatchPAT 300 device. These devices include a wrist device that uses a plethysmography-based finger-mounted probe that measures the PAT signal and oxygen saturation, which is used as a proxy for respiratory disturbances and overnight sleep. A chest sensor records snoring, body position, and chest movement signals. The WatchPAT-ONE incorporates the same algorithm and technology as the WatchPAT 300, which exerted high sensitivity (85–89%) and fair specificity (63–77%) when compared to polysomnography (PSG) for diagnosing sleep apnoea [9–11]. WatchPAT was specifically validated in AF patients [10].

A minimum of 4 h valid recording time with the WatchPAT device was required. WatchPAT data were analysed by a validated algorithm and reviewed by a certified sleep physician according to methods described in the American Academy of Sleep Medicine manual for the scoring of sleep and associated events [12]. The WatchPAT device detects respiratory events by the detection of sympathetic activations and concomitant oxygen desaturations. The WatchPAT-derived apnoea–hypopnoea index (pAHI) was calculated as the total number of apnoeas plus hypopnoeas divided by the total sleep time in hours. The apnoea-severity was determined according to the following pAHI categories: pAHI 5–15: mild SDB; pAHI 15–30: moderate SDB; pAHI ≥ 30: severe SDB; pAHI ≥ 15: moderate-to-severe SDB. In the current study, moderate-to-severe SDB was considered to be clinically relevant SDB.

### STOP-Bang questionnaire

The STOP-Bang questionnaire is a validated and widely used screening tool for SDB [2, 7]. The questionnaire consists of eight dichotomous questions (S, Snoring, T, tiredness, O, observed apnoeas, P, high blood pressure, B, body mass index (BMI) ≥ 35 kg/m^2^, A, age > 50 years, N, neck circumference > 40 cm, and G, male gender) [13]. Each positive answer is assigned one point. The sum of these points determines the risk of moderate-to-severe SDB: patients with a score of 0–2 are classified as having a low risk of SDB, patients with a score of 3–4 as having an intermediate to high risk, and patients with a score of 5–8 as having a high risk of SDB. For the current study, a score of ≥ 3 (intermediate to high risk of SDB) was considered as a positive test. Study participants completed the questionnaire digitally upon entry in the study.

### Improving pre-selection with the BOSS-GAP score

Even though the STOP-Bang questionnaire is one of the most frequently used SDB screening tools, previous studies indicate a limited validity to detect SDB in patients with AF [6, 7]. Next to assessing the accuracy of STOP-Bang questionnaire in detecting moderate-to-severe SDB in our cohort, we aimed to develop a refined, AF-specific SDB screening tool based on the existing STOP-Bang questionnaire combined with additional patient characteristics. To develop this score, consecutive groups of patients were divided into a training (*n* = 106) and a validation cohort (*n* = 100). Within the training cohort, STOP-Bang items were included in multivariable logistic regression. Those STOP-Bang items with beta-coefficients of < 0.05 or negative correlation in multivariable logistic regression were considered of limited additional value to the endpoint and were removed from the AF-specific score, while STOP-Bang items associated with moderate-to-severe SDB remained. Moreover, clinical variables associated with the presence of moderate-to-severe SDB in univariable analyses were included in a multivariable regression analysis. Variables with a significant association (*α* = 0.1) in this multivariable analysis were added to the remaining STOP-Bang items to create an AF-specific score. Optimal cut-off points for continuous variables (age and BMI) were determined as the point maximizing the Youden’s index. The calibrated beta-coefficients from the multivariable model were used to derive a clinical point-based scoring system, with the lowest coefficient as a denominator. The performance of the resulting score was assessed in the validation cohort.

### Statistical analyses

Continuous variables were tested for normality with the Kolmogorov–Smirnov test and by visual interpretation. Variables with normal distribution were expressed as mean ± standard deviation (SD) and compared using the unpaired *t*-test, nonparametric variables were expressed as median with interquartile range (IQR) and compared using the Mann–Whitney *U* test. Categorical variables were presented as counts (*n*) with percentages (%) and compared using the *χ*^2^ test or Fisher’s Exact test, whichever is appropriate. Spearman correlations were performed to assess correlation between components of the STOP-Bang questionnaire.

The sensitivity, specificity, positive predictive value (PPV), negative predictive value (NPV), and Cohen’s kappa were calculated for different cut-off points of screening tools to assess their capability to detect moderate-to-severe SDB (pAHI ≥ 15). The predictive performance of screening tools was evaluated by calculating the area under the receiver operating characteristic (ROC) curves (AUROC). For the new AF-specific score, separate ROC curves were constructed for the training (*n* = 106), validation (*n* = 100) and total (*n* = 206) cohort. Screening tools were considered to perform well when the AUROC exceeded 0.7. In addition, calibration was evaluated (Spiegelhalter *z* test), net reclassification improvement (NRI) and integrated discrimination improvement (IDI) were calculated. Reclassification was further assessed in a reclassification table. Decision curve analysis was performed to compare net benefit of using the STOP-Bang and the new AF-specific score as pre-selection tools.

A two-sided *P* value of 0.05 was considered statistically significant. Statistical analyses were performed using IBM SPSS Statistics software (version 25.0, IBM Corp., Amonk, NY, USA) and SAS^®^.

## Results

A total of 268 patients completed SDB screening via the Virtual-SAFARI management pathway at the time of the current analysis. Of those, 62 were excluded due to failure to complete the STOP-Bang questionnaire (*n* = 36) or failure to complete it within six months of SDB screening (*n* = 26). The remaining 206 patients (77%) were included in the current study.

### Study population

In this cohort of AF patients awaiting CA (58% male, median age 65 [58–70] years), prevalence of moderate-to-severe SDB was 51%, *n* = 106. Mild, moderate and severe SDB were newly diagnosed in 70 (34%), 71 (34%), and 35 (17%) patients, respectively. Patients’ characteristics are presented in Table 1. Patients with moderate-to-severe SDB had a higher BMI (28 [26–31] vs. 26 [24–29] kg/m^2^, *P* < 0.001), higher thromboembolic risk (CHA_2_DS_2_-VASc score 2 [1–3] vs. 2 [1–3], *P* = 0.022), more often had hypertension (53% vs. 39%, *P* = 0.047), dyslipidaemia (24% vs. 11%, *P* = 0.017), previous thromboembolic events (17% vs. 3%, *P* = 0.002), or vascular disease (10% vs. 20%, *P* = 0.049), and were more often prescribed vitamin K antagonists (0% vs. 4%, *P* = 0.050) compared to those with none or mild SDB.

**Table 1:** Baseline characteristics of the entire cohort (*n* = 206) and comparison between different groups of severity of sleep disordered breathing

Variable	Overall (*n* = 206)	None and mild SDB (*n* = 100)		Moderate-to-severe SDB (*n* = 106)		*P* value^1^
		None (pAHI < 5) (*n* = 30)	Mild (pAHI 5 to < 15) (*n* = 70)	Moderate (pAHI 15 to < 30) (*n* = 71)	Severe (pAHI ≥ 30) (*n* = 35)	
Demographics						
Age, years	65 [58–70]	65 [55–70]		65 [61–71]		0.101
		60 [50–69]	66 [59–70]	65 [60–70]	64 [61–73]	
Male	120 (58%)	9 (30%)	43 (61%)	47 (66%)	21 (60%)	0.077
BMI, kg/m^2^	27 [25–30]	26 [24–29]		28 [26–31]		< 0.001
		25 [23–27]^2^	27 [24–30]^2^	27 [25–30]^3^	31 [28–33]^3^	
BMI ≥ 27 kg/m^2^	108 (52%)	6 (20%)^2^	36 (51%)^2^	37 (52%)^3^	29 (83%)^3^	0.004
AF characteristics						
Paroxysmal AF	139 (68%)	25 (83%)	44 (63%)	49 (70%)	21 (60%)	0.721
EHRA I	3 (2%)	1 (3%)	0 (0%)	2 (3%)	0 (0%)	0.650
EHRA II	130 (63%)	18 (60%)	48 (69%)	43 (61%)	21 (60%)	
EHRA III	73 (35%)	11 (37%)	22 (31%)	26 (37%)	14 (40%)	
CHA_2_DS_2_-VASc score	2 [1–3]	2 [1–3]		2 [1–3]		0.022
		1 [1–2]	2 [1–3]	2 [1–3]	2 [1–3]	
Comorbidities and risk factors						
Dyslipidaemia	36 (18%)	1 (3%)	10 (14%)	17 (24%)	8 (23%)	0.017
Diabetes mellitus	19 (9%)	3 (10%)	5 (7%)	7 (10%)	4 (11%)	0.556
Congestive heart failure	32 (16%)	2 (7%)	9 (13%)	14 (20%)	7 (20%)	0.081
Vascular disease	31 (15%)	0 (0%)^2^	10 (14%)^2^	14 (20%)	7 (20%)	0.049
Previous stroke or TIA	20 (10%	0 (0%)	3 (4%)	11 (15%)	6 (17%)	0.002
Smoking						
Actively	21 (10%)	0 (0%)	7 (10%)	8 (12%)	6 (17%)	0.525
Previously	59 (29%)	7 (23%)	23 (33%)	22 (32%)	7 (20%)	
Never	123 (61%); *n* = 203	23 (77%)	39 (57%); *n* = 69	39 (57%); *n* = 69	22 (63%); *n* = 35	
Alcohol consumption						
None	50 (25%)	9 (30%)	17 (25%)	14 (21%)	10 (29%)	0.551
< 5 units/week	99 (49%)	13 (43%)	38 (55%)	29 (43%)	19 (59%)	
5–15 units/week	47 (23%)	8 (27%)	12 (17%)	24 (35%)	3 (8.8%)	
> 15 units/week	5 (2.5%) *n* = 201	0 (0%)	2 (2.9%) *n* = 69	1 (1.5%) *n* = 68	2 (5.9%) *n* = 34	
STOP-Bang components (reported by patients)						
Snoring	51 (25%)	0 (0%)^2^	17 (24%)^2^	20 (28%)	14 (40%)	0.012
Tiredness	118 (57%)	17 (57%)	43 (61%)	32 (45%)^3^	26 (74%)^3^	0.444
Observed apnoeas	50 (24%)	3 (10%)	12 (17%)	23 (32%)	12 (34%)	0.003
High blood pressure	103 (50%)	9 (30%)	36 (51%)	36 (51%)	22 (63%)	0.163
BMI > 35 kg/m^2^	26 (13%)	3 (10%)	5 (7%)	8 (11%)	10 (29%)	0.052
Age > 50 years	186 (90%)	22 (73%)	61 (87%)	71 (100%)^3^	32 (91%)^3^	0.001
Neck circumference > 40 cm	52 (25%)	1 (3.3%)^2^	22 (31%)^2^	16 (23%)	13 (37%)	0.472
Male	122 (59%)	10 (33%)^2^	43 (61%)^2^	48 (68%)	21 (35%)	0.077
Available STOP-Bang components (based on electronic health records)						
Hypertension	95 (46%)	7 (23%)^2^	32 (46%)^2^	36 (51%)	20 (57%)	0.047
BMI > 35 kg/m^2^	9 (4%)	0 (0%)	4 (6%)	3 (4%)	2 (6%)	0.801
Age > 50 years	188 (91%)	22 (73%)2	61 (87%)^2^	71 (100%)	34 (97%)	< 0.001
Male	120 (58%)	9 (30%)^2^	43 (61%)^2^	47 (66%)	21 (60%)	0.077
Cardiovascular drugs						
Beta-blockers	93 (45%)	12 (40%)	29 (41%)	35 (49%)	17 (49%)	0.246
Digitalis	15 (7%)	4 (13%)	4 (6%)	4 (6%)	3 (9%)	0.700
Antiarrhythmic drugs	127 (62%)	19 (63%)	39 (56%)	47 (66%)	22 (63%)	0.295
VKA	4 (2%)	0 (0%)	0 (0%)	4 (6%)	0 (0%)	0.050
NOAC	192 (93%); *n* = 205	28 (93%)	63 (90%) *n* = 69	66 (93%)	35 (100%)	0.361

Number provided after the semicolon indicates the total number of patients available for that variable

*AF* atrial fibrillation, *AHI* apnoea–hypopnea index, *BMI* body mass index, *EHRA* European Heart Rhythm Association, *NOAC* non-vitamin K antagonist oral anticoagulant, *SDB* sleep disordered breathing, *TIA* Transient Ischemic Attack *VKA* vitamin K antagonist

^1^*P* values are given for comparison between none-to-mild vs. moderate-to-severe SDB

^2^Represents statistically significant differences between none vs mild SBD (BMI: *P* = 0.013, BMI ≥ 27 kg/m^2^: *P* = 0.004, hypertension: *P* = 0.035, vascular disease *P* = 0.030; age > 50 years: *P* = 0.044)

^3^Represents statistically significant differences between moderate vs severe SDB (BMI: *P* = 0.001, BMI ≥ 27 kg/m^2^: *P* = 0.002)

### Performance of the STOP-Bang questionnaire

**Fig. 1:**
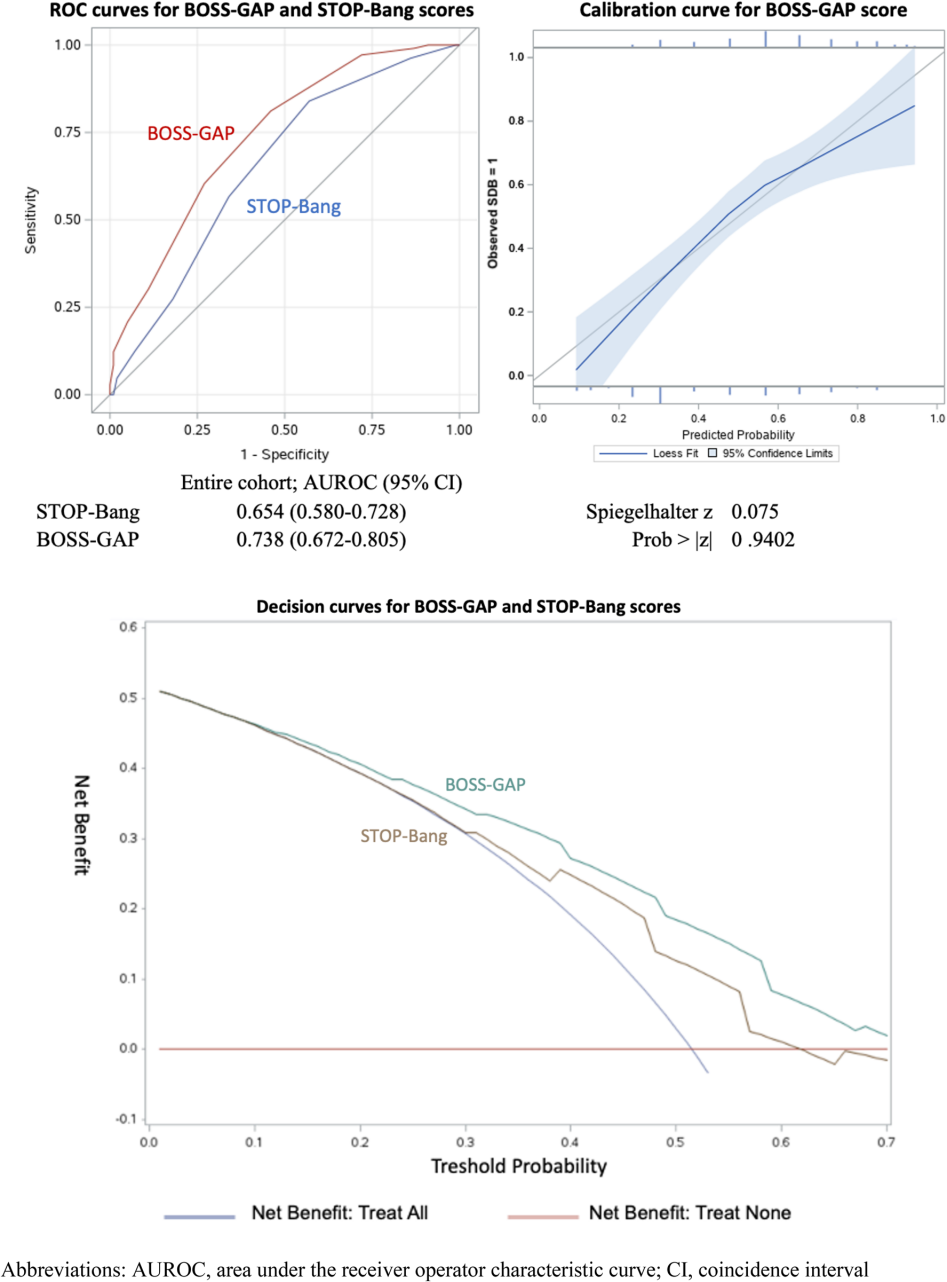
Receiver operating characteristic curve, calibration curve and decision curve analyses of models to predict moderate-to-severe sleep disordered breathing (*n* = 206). *AUROC* area under the receiver operator characteristic curve, *CI* confidence interval

The median STOP-Bang score in the overall study population was 3 [2–4]. The STOP-Bang questionnaire performed poorly as a screening tool for moderate-to-severe SDB (AUROC 0.654, 95% CI 0.580–0.728, Fig. 1). The sensitivity, specificity, PPV and NPV for different STOP-Bang scores are provided in Table 2. The most frequently used cut-off point for the STOP-Bang questionnaire, ≥ 3 points, demonstrated a sensitivity of 84% and a NPV of 72%.

**Table 2:** Sensitivity, specificity, positive predictive value, negative predictive value and kappa for different cut-off points of the STOP-Bang questionnaire and the BOSS-GAP score to predict moderate-to-severe sleep disordered breathing

Cut-off point	Sensitivity	Specificity	PPV	NPV
STOP-Bang (patient reported)				
≥ 2	96% (102/106)	14% (14/100)	54% (102/188)	78% (14/18)
≥ 3 (moderate to high risk)	84% (89/106)	43% (43/100)	61% (89/146)	72% (43/60)
≥ 5 (high risk)	27% (29/106)	82% (82/100)	62% (29/47)	52% (82/159)
STOP-Bang (according to EHR)				
≥ 2	98% (104/106)	15% (15/100)	55% (104/189)	88% (13/17)
≥ 3 (moderate to high risk)	81% (86/106)	46% (46/100)	61% (86/140)	70% (46/66)
≥ 5 (high risk)	25% (26/106)	84% (84/100)	62% (26/42)	51% (84/164)
BOSS-GAP				
≥ 4 (moderate to high risk)	97% (103/106)	24% (24/100)	58% (103/179)	89% (24/27)
≥ 6 (high risk)	81% (86/106)	55% (55/100)	66% (86/131)	73% (55/75)

*EHR* electronic health records, *NPV* negative predictive value, *PPV* positive predictive value

When comparing patient-reported STOP-Bang items and items derived from electronic health records (EHR) (hypertension, BMI, age, and sex were available), some misclassifications were found (*n* = 62, 7.4%). Substituting patient-reported variables with results derived from EHR did not impact STOP-Bang performance (sensitivity for cut-off point ≥ 3 81%, NPV 70%, AUROC 0.647, 95% CI 0.573–0.721), as described in Table 2.

**Fig. 2:**
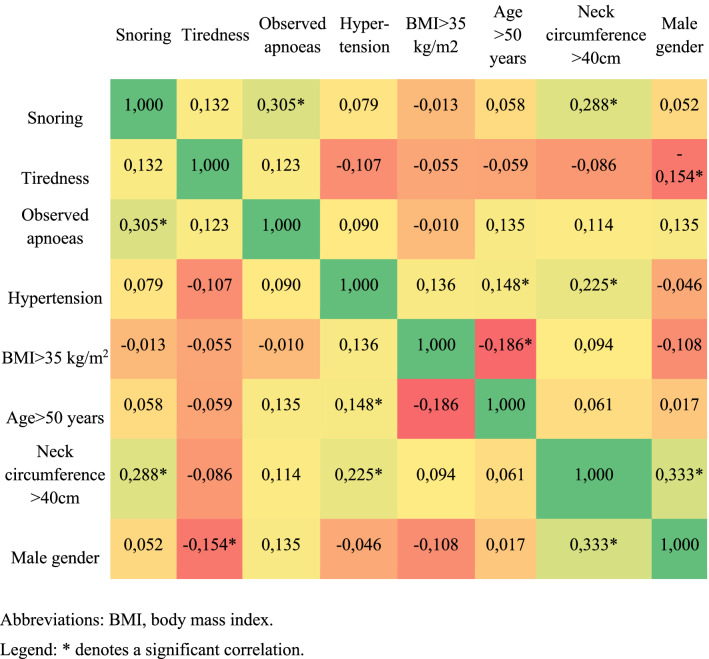
Spearman correlation between STOP-Bang components. *BMI* body mass index. * denotes a significant correlation

Several correlations between individual STOP-Bang components were observed. Components positively correlated were snoring and observed apnoeas or neck circumference > 40 cm, high blood pressure or hypertension and neck circumference > 40 cm, and male gender and neck circumference > 40 cm. Components negatively correlated were age > 50 years and BMI > 35 kg/m^2^, tiredness and male gender (Fig. 2).

### AF-specific refinement of the STOP-Bang questionnaire: the BOSS-GAP score

**Fig. 3:**
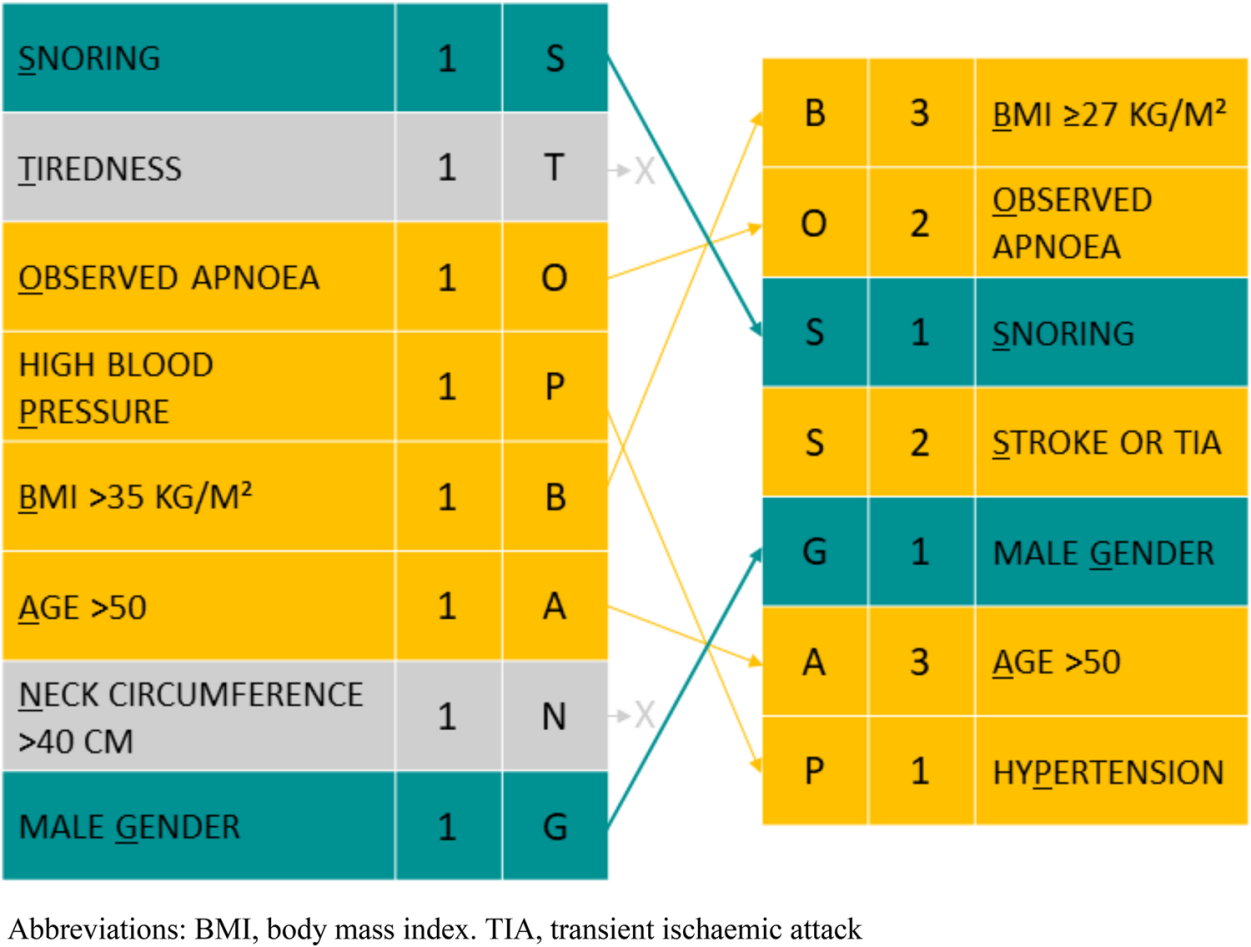
STOP-Bang and BOSS-GAP scores. *BMI* body mass index, *TIA* transient ischaemic attack. The original STOP-Bang score and the new, AF-specific BOSS-GAP score. STOP-Bang variables that were removed from the AF-specific score (not included in BOSS-GAP) are shown in grey. Green variables were included in the BOSS-GAP score without any modifications. Yellow variables were either newly added to the BOSS-GAP score, or additional points were assigned based on multivariable regression coefficients

The training cohort used to derive the AF-specific SDB score consisted of 106 patients. There were no significant differences in baseline characteristics between the training- and validation cohort (Supplemental Table S1). In the training cohort, uni-, and multivariable regression analyses revealed that neck circumference and tiredness were not positively correlated with moderate-to-severe SDB (Supplemental Tables S2 and S3). These variables were removed from the refined AF-specific score. Several baseline characteristics were associated with moderate-to-severe SDB (Supplemental Table S2). After multivariable regression analysis, the following variables remained important: BMI (optimal cut-off point ≥ 27 kg/m^2^), age (optimal cut-off point > 50 years old, corresponding with the existing STOP-Bang item), snoring, observed apnoeas, previous stroke or transient ischemic attack (TIA), hypertension and male gender. BMI with cut-off point ≥ 27 kg/m^2^ and previous stroke or TIA were added to the new AF-specific SDB screening score. Based on the beta coefficients in the multivariable regression model, highest points (3) were assigned for age > 50 years and BMI ≥ 27 kg/m^2^, and two points were assigned for observed apnoea and previous stroke or TIA (Supplemental Table S4). The remaining items received one point. This approach resulted in the BOSS-GAP score (Fig. 3).

In the training cohort of 106 patients, the BOSS-GAP score demonstrated a good predictive power in estimating the risk of moderate-to-severe SDB, with an AUROC of 0.803 (95% CI 0.721–0.885) (Supplemental Fig. S1). Calibration plots graphically showed good agreement (*P* = 0.961 for the Spiegelhalter’s Z-test) on the presence of moderate-to-severe SDB between the risk estimation by the BOSS-GAP and WatchPAT confirmation.

In the validation cohort, the BOSS-GAP displayed an AUROC of 0.673 (95% CI 0.568–0.778) for the estimation of moderate-to-severe SDB risk. The observed frequencies and the estimated probability of moderate-to-severe SDB presence showed a good calibration curve (*P* = 0.955 for the Spiegelhalter’s *Z*-test) for risk estimation (Supplemental Fig. S1). In the overall study population, the score displayed an AUROC of 0.738 (95% CI 0.672–0.805) (Fig. 1). The category free net reclassification improvement (NRI) for the new BOSS-GAP Score compared to STOP-Bang results for moderate-to-severe SDB was 0.201 (0.085–0.917, *P* < 0.05) and integrated discrimination improvement was 1.35 (0.885–1.815, *P* < 0.001) in the overall cohort, respectively.

### Clinical value of the STOP-Bang and BOSS-GAP score in pre-selection for SDB screening

The STOP-Bang questionnaire was able to correctly identify 89 (84%) of confirmed SDB patients as at risk for SDB (cut-off value ≥ 3). If STOP-Bang would have been used as a pre-selection tool to determine which patients should be referred for SDB screening in our cohort of AF patients, a total of 60 (29%) of patients would not have been referred for screening. However, this would have led to missed diagnoses of SDB cases in 17 (8%) of patients.

The refined AF specific BOSS-GAP score (cut-off value ≥ 4) correctly marked 103 (97%) of SDB patients as being at risk. A total of 25 (12%) of patients had a negative BOSS-GAP score, hence, this proportion of WatchPAT referrals could have been prevented. Since only three of these patients (1.5%) had SDB, omitting SDB screening for patients with a BOSS-GAP score below 4 may be considered with low risk of missing SDB cases (Supplementary Table S5).

Decision curves analysis (Fig. 1) suggests that using the STOP-Bang or BOSS-GAP scores as preselection tools might be useful for threshold probabilities above 30% for STOP-Bang and 12% for BOSS-GAP. The BOSS-GAP score had higher net benefit than STOP-Bang across the range of threshold probabilities. However, as WatchPAT is not an invasive test, for most patients lower threshold probabilities may be acceptable (i.e. the number of patients needed to screen with WatchPAT to detect one case of SDB may be higher than 8, corresponding with a threshold probability of 12.5%). In the lower range of threshold probabilities (< 12%), both the BOSS-GAP and the STOP-Bang curves overlap with the ‘treat all’ line. Therefore, for lower threshold probabilities both questionnaires provide no net benefit over structural WatchPAT screening.

## Discussion

In the current study, we demonstrated that the STOP-Bang questionnaire performed poorly to pre-select AF patients at risk of SDB. Being used as a pre-selection tool, a high proportion of patients with SDB would have been classified as false negative for risk of SDB. However, we demonstrated that a refined version of the STOP-Bang score, the BOSS-GAP score, performed better in our cohort of AF patients scheduled for CA. In the overall cohort, using this score as a pre-selection tool to determine which patients should be referred for SDB screening had the potential to save one in six home sleep tests, with a low probability to miss SDB cases.

This study is not the first to demonstrate the limited value of the STOP-Bang questionnaire as an SDB-screening tool in AF patients. Several studies reported its moderate to poor performance in detecting moderate-to-severe SDB in AF patients in different clinical settings [6, 7, 14–16]. The relatively high false negative rate and low sensitivity have been mentioned as factors limiting the usefulness of the STOP-Bang questionnaire [17]. However, causes for this limited usability in AF patients may be unrelated to the questionnaire items themselves [6]. Originally, the questionnaire was not developed specifically for AF patients. Due to interrelations between SDB and AF, the shared risk factor profile, and the fact that symptoms of SDB and AF are often overlapping, the STOP-Bang items might have limited predictive value [6, 7, 14–19]. Indeed, in our cohort, some overlapping characteristics could be revealed with correlation analysis of the STOP-Bang items, for example, between the item neck circumference and observed apnoea/snoring, high blood pressure or male gender. However, when further analysing the different STOP-Bang items, the item neck circumference did not appear to be a predictive characteristic for SDB presence in our cohort.

Another explanation of the poor performance of the STOP-Bang questionnaire is the subjective nature of some of its items [6]. For example, SDB patients with AF report lower daytime sleepiness than those without AF [20] and other STOP-Bang items (snoring, observed apnoea) are self-reported, while we noticed an inaccuracy in STOP-Bang characteristics when comparing self-reported items to those derived from EHR. This is in line with a previous study reporting on large differences in patient-reported risk factors, patient characteristics and CHA_2_DS_2_-VASc score compared to these factors assessed by healthcare professionals [21]. However, substituting patient-reported STOP-Bang items with data from the EHR did not change the overall performance of the STOP-Bang questionnaire in our cohort.

After refinement of the STOP-Bang questionnaire in our cohort, the STOP-Bang items neck circumference and tiredness were not associated with an elevated chance of moderate-to-severe SDB but having had a previous stroke or TIA (which is also included in the CHA_2_DS_2_-VASc score) was. The refined STOP-Bang-based BOSS-GAP score showed a stronger discriminatory performance to identify patients with increased risk of moderate-to-severe SDB than the STOP-Bang questionnaire. However, even after refinement of the screening questionnaire, no good performance (AUROC > 0.7) could be achieved in the validation cohort. Further studies are required to determine the clinical relevance of the refined score. The value of pre-selection for SDB screening in patients with AF based on questionnaires might therefore still be limited.

### Implementation of AF-specific pre-selection tools for SDB screening

SDB remains a highly prevalent comorbidity in AF and SDB screening and management is mentioned as an important component of a combined risk factor management program. However, official recommendations and practical guides for implementation of systematic SDB screening in AF patients scheduled for CA are missing [2, 7, 22]. An integrated AF care approach with a multidisciplinary, patient-focused collaboration between sleep physicians and AF teams has been proposed [2, 22, 23] and implemented within the Virtual-SAFARI project [5]. However, several barriers might appear in the implementation of systematic SDB testing, such as a lack of skills and knowledge, financial and workforce-related resources, and missing collaboration between cardiology and sleep medicine, as described by the EHRA and ACNAP survey [4]. Incorporating questionnaires or scoring systems, such as the BOSS-GAP score, into EHR [6], so that risk scores are automatically calculated, may simplify pre-selection of patients who should be referred to SDB management pathways. However, our findings indicate that adequate pre-selection for SDB screening in patients with AF, especially by using questionnaires, remains challenging. Available safe and easy options such as ambulatory, systematic screening approaches might therefore still be the best solution. In the future, incorporation of data from wearable devices, smartphone apps and cardiac implantable devices in the pre-selection process may help to refine identification of those patients requiring SDB assessment further, which may lead to easier identification of patients at risk of SDB.

### Implications for future research and clinical practice

When compared to systematic SDB screening in patients with AF, pre-selection of patients at higher risk for SDB could reduce the number of patients who are referred for SDB screening. However, the current results should first be validated to assess whether the newly developed BOSS-GAP performs consistently in other AF populations. Furthermore, future research is needed regarding cost-efficacy of pre-selection methods such as proposed in our study. Additionally, further research is needed towards the optimal method for dissemination and broad implementation of the score. This could be achieved by implementing a BOSS-GAP score-based pre-selection as part of an integrated AF and SDB management pathway in patients scheduled for CA, as previously proposed and implemented [2, 5, 23].

### Limitations

Our study has several limitations. Firstly, SDB diagnosis was based on an overnight home sleep test with the WatchPAT device, and not on PSG. This together with the fact that the screening is based on a single measurement, which does not consider possible night-to-night variations of SDB, may influence the SDB diagnosis. Secondly, although WatchPAT-ONE uses the same algorithm and sensor technology as WatchPAT 300 which has been validated in AF patients, studies using the WatchPAT-ONE device are limited. Thirdly, patients with known SDB or previous SDB screening were excluded from our study, which might influence pre-test probability in our study cohort. However, previous studies assessing SDB prevalence in AF patients in different clinical settings report equally high percentages. Fourthly, the digital SDB management pathway was tested in AF patients scheduled for CA only and we implemented the digital pathway in only two centres in the Netherlands, where inclusion of patients with a BMI > 35 kg/m^2^ was limited. Finally, the BOSS-GAP was based on a relatively small training group and validated in a small group as well. Therefore, it requires validation in a larger, preferably external cohort.

## Conclusions

In our cohort of consecutive patients scheduled for AF CA, the STOP-Bang questionnaire showed limited value when used as a pre-selection tool for SDB screening. The AF-specific refinement of the STOP-Bang questionnaire resulted in the novel BOSS-GAP questionnaire which demonstrated slightly improved, but still limited accuracy in identifying AF patients with moderate-to-severe SDB. Whether questionnaires bring an advantage regarding pre-selection for SDB screening compared to systematic screening in all patients with AF, requires further larger studies.

## Supplementary Information

Below is the link to the electronic supplementary material.

Supplementary file1 (DOCX 134 KB)

## Data Availability

The datasets generated and analysed during the current study are not publicly available due possible compromise of privacy but are available from the corresponding author on reasonable request.
