# Supplementary material for: Atrial fibrillation-specific refinement of the STOP-Bang sleep apnoea screening questionnaire: insights from the Virtual-SAFARI study
Source: Clin Res Cardiol. 2023 Feb 11;112(6):834–45. doi: 10.1007/s00392-023-02157-9 (PMC10241725; doi:10.1007/s00392-023-02157-9)
Supplement: Supplementary file 1 — Supplementary file1 (DOCX 134 KB) [file 392_2023_2157_MOESM1_ESM.docx]

**Supplementary material to the manuscript “Atrial fibrillation-specific refinement of the STOP-Bang sleep apnoea screening questionnaire: Insights from the Virtual-SAFARI study.”**

Konstanze Betz^1,2^*, Dominique Verhaert^1,3^*, Monika Gawalko^1,4,5^*, Astrid Hermans^1^, Zarina Habibi,^1,3^ Nikki A.H.A. Pluymaekers,^1^ Rachel van der Velden,^1^ Marloes Homberg,^6^ Suzanne Philippens^1^, Maartje J.M. Hereijgers^1^, Bianca Vorstermans^1^, Sami O. Simons^1,7^, Dennis den Uijl^1^, Sevasti-Maria Chaldoupi^1^, Justin Luermans^1^, Sjoerd Westra^3^, Theo Lankveld^1^, Reindert van Steenwijk^8^, Bernard Hol^8^, Ulrich Schotten^1^, Kevin Vernooy^1^, Jeroen M Hendriks^9,10+^, Dominik Linz^1,3,9,10,11+^.

^1^ Department of Cardiology, Cardiovascular Research Institute Maastricht (CARIM), Maastricht University Medical Centre, P. Debyelaan 25, 6229 HX Maastricht, the Netherlands

^2^ Department of Internal Medicine, Eifelklinik St. Brigida GmbH & Co. KG, Kammerbruchstraße 8, 52152 Simmerath, Germany

^3^ Department of Cardiology, Radboud University Medical Center and Radboud Institute for Health Sciences, Geert Grooteplein Zuid 10, 6525 GA Nijmegen, the Netherlands

^4^ 1st Department of Cardiology, Doctoral School, Medical University of Warsaw, Żwirki i Wigury 61, 02-091 Warsaw, Poland

^5^ Institute of Pharmacology, West German Heart and Vascular Centre, University Duisburg-Essen, Forsthausweg 2, 47057 Duisburg, Germany

^6^ Department of Anesthesiology, Maastricht University Medical Center, P. Debyelaan 25, 6229 HX Maastricht, the Netherlands

^7^ Department of Respiratory Medicine, Maastricht University Medical Centre, P. Debyelaan 25, 6229 HX Maastricht, the Netherlands

^8^ Netherland Sleep Institute, Computerweg 4, 3821 AB Amersfoort, the Netherlands

^9^ Centre for Heart Rhythm Disorders, University of Adelaide and Royal Adelaide Hospital, Port Rd, SA 5000 Adelaide, Australia

^10^ Caring Futures Institute, College of Nursing and Health Sciences, Flinders University, Sturt Rd, Bedford Park SA 5042, Adelaide, Australia

^11^ Department of Biomedical Sciences, Faculty of Health and Medical Sciences, University of Copenhagen, Nørregade 10, 1165 Copenhagen, Denmark

Address for Correspondence:

Dominik Linz, MD, PhD

Maastricht UMC+

Maastricht Heart+Vascular Center

6202 AZ Maastricht

E [dominik.linz@mumc.nl](mailto:dominik.linz@mumc.nl) | T +31(0)43-3875093 | M +31(0)6-123 99 1

**Supplementary Table S1.** Comparison of baseline characteristics between training (n=106) and validation (n=100) cohorts.

| **Variable** | **Training cohort**  **(n=106)** | **Validation cohort (n=100)** | ***P* value** |
| --- | --- | --- | --- |
| **Demographics (based on electronic health records)** | | | |
| **Age, years** | 66 [59-73] | 64 [58-69] | 0.139 |
| **Age >50 years** | 97 (92%) | 91 (91%) | 0.897 |
| **Male** | 58 (55%) | 62 (62%) | 0.289 |
| **BMI, kg/m^2^** | 27 [25-31] | 27 [25-30] | 0.444 |
| **BMI** ≥ **27 kg/m^2^** | 56 (53%) | 52 (52%) | 0.508 |
| **BMI** > **35 kg/m^2^** | 6 (6%) | 3 (3%) | 0.350 |
| **AF characteristics (based on electronic health records)** | | | |
| **Paroxysmal AF** | 74 (70%) | 65 (66%) | 0.525 |
| **EHRA I**  **EHRA II**  **EHRA III** | 3 (2.8%)  60 (57%)  43 (41%) | 0 (0%)  70 (70%)  30 (30%) | 0.052 |
| **CHA₂DS₂-VASc score** | 2 [1-3] | 2 [1-3] | 0.312 |
| **Comorbidities and risk factors (based on electronic health records)** | | | |
| **Hypertension** | 50 (47%) | 45 (45%) | 0.755 |
| **Dyslipidemia** | 17 (16%) | 19 (19%) | 0.576 |
| **Diabetes mellitus** | 9 (8%) | 10 (10%) | 0.708 |
| **Congestive heart failure** | 17 (16%) | 15 (15%) | 0.837 |
| **Vascular disease** | 13 (12%) | 18 (18%) | 0.250 |
| **Previous stroke/TIA** | 1 (10%) | 9 (9%) | 0.739 |
| **Smoking**  Actively  Previously  Never | 12 (11%)  32 (31%)  61 (58%)  N=105 | 9 (9.2%)  27 (28%)  62 (63%)  N=98 | 0.885 |
| **Alcohol consumption**  None  <5 units/week  5-15 units/week  >15 units/week | 25 (24%)  50 (49%)  27 (26%)  1 (1.0%)  N=103 | 25 (26%)  49 (50%)  20 (20%)  4 (4.1%)  N=98 | 0.435 |
| **STOP-Bang components (patient reported)** | | | |
| **Snoring** | 28 (26%) | 23 (23%) | 0.570 |
| **Tiredness** | 59 (56%) | 59 (59%) | 0.628 |
| **Observed apnoeas** | 22 (21%) | 28 (28%) | 0.225 |
| **High blood pressure** | 52 (49%) | 51 (51%) | 0.780 |
| **BMI** >**35 kg/m^2^** | 15 (14%) | 11 (11%) | 0.496 |
| **Age >50 years** | 97 (92%) | 89 (89%) | 0.543 |
| **Neck circumference >40cm** | 30 (28%) | 22 (22%) | 0.298 |
| **Male** | 58 (55%) | 64 (64%) | 0.175 |
| **Cardiovascular drugs (based on electronic health records)** | | | |
| **Beta-blockers** | 49 (46%) | 44 (44%) | 0.781 |
| **Digitalis** | 9 (8.5%) | 6 (6.0%) | 0.596 |
| **Antiarrhythmic drugs** | 65 (61%) | 62 (62%) | 1.000 |
| **VKA** | 3 (2.8%) | 1 (1.0%) | 0.622 |
| **NOAC** | 97 (92%) | 95 (96%)  N=99 | 0.255 |

**Abbreviations**: AF, atrial fibrillation; BMI, body mass index; EHRA, European Heart Rhythm Association; NOAC, non-vitamin K antagonist oral anticoagulant; TIA; transient ischaemic attack; VKA, vitamin K antagonist

**Supplementary Table S2.** Comparison of baseline characteristics and STOP-Bang components within the training cohort (n=106).

| **Variable** | **None and mild SDB (n= 50)** | **Moderate-to-severe SDB (n=56)** | ***P* value** |
| --- | --- | --- | --- |
| **Demographics (based on electronic health records)** | | | |
| **Age, years** | 65 [56-70] | 66 [61-74] | 0.053 |
| **Age >50 years** | 41 (82%) | 56 (100%) | 0.001 |
| **Male** | 24 (48%) | 34 (61%) | 0.189 |
| **BMI, kg/m^2^** | 26 [24-29] | 29 [26-32] | 0.001 |
| **BMI** ≥ **27 kg/m^2^** | 18 (36%) | 38 (68%) | 0.001 |
| **BMI** > **35 kg/m^2^** | 4 (8%) | 2 (4%) | 0.325 |
| **AF characteristics (based on electronic health records)** | | | |
| **Paroxysmal AF** | 35 (70%) | 39 (70%) | 0.968 |
| **EHRA I**  **EHRA II**  **EHRA III** | 1 (2%)  31 (62%)  18 (35%) | 2 (4%)  29 (52%)  25 (45%) | 0.548 |
| **CHA₂DS₂-VASc score** | 2 [1-3] | 2 [1-3] | 0.034 |
| **Comorbidities and risk factors (based on electronic health records)** | | | |
| **Hypertension** | 19 (38%) | 31 (55%) | 0.074 |
| **Dyslipidemia** | 5 (10%) | 12 (21%) | 0.109 |
| **Diabetes mellitus** | 4 (8%) | 5 (9%) | 0.864 |
| **Congestive heart failure** | 4 (8%) | 13 (23%) | 0.033 |
| **Vascular disease** | 4 (8%) | 9 (16%) | 0.206 |
| **Previous stroke/TIA** | 1 (2%) | 10 (18%) | 0.008 |
| **Smoking**  Actively  Previously  Never | 3 (6.0%)  15 (26%)  32 (64%) | 9 (16%)  17 (31%)  29 (53%)  N=55 | 0.141 |
| **Alcohol consumption**  None  <5 units/week  5-15 units/week  >15 units/week | 13 (26%)  26 (52%)  11 (22%)  0 (0%) | 12 (23%)  24 (45%)  16 (30%)  1 (1.9%)  N=53 | 0.581 |
| **STOP-Bang components (patient reported)** | | | |
| **Snoring** | 10 (20%) | 18 (32%) | 0.157 |
| **Tiredness** | 28 (56%) | 31 (55%) | 0.947 |
| **Observed apnoeas** | 5 (10%) | 17 (30%) | 0.010 |
| **High blood pressure** | 22 (44%) | 30 (54%) | 0.325 |
| **BMI** >**35 kg/m^2^** | 4 (8%) | 11 (20%) | 0.086 |
| **Age >50 years** | 41 (82%) | 56 (100%) | 0.001 |
| **Neck circumference >40cm** | 11 (22%) | 19 (34%) | 0.174 |
| **Male** | 24 (48%) | 34 (61%) | 0.189 |
| **Cardiovascular drugs (based on electronic health records)** | | | |
| **Beta-blockers** | 18 (36%) | 31 (55%) | 0.053 |
| **Digitalis** | 4 (8.0%) | 5 (8.9%) | 1.000 |
| **Antiarrhythmic drugs** | 30 (60%) | 35 (63%) | 0.843 |
| **VKA** | 0 (0%) | 3 (5.4%) | 0.245 |
| **NOAC** | 45 (90%) | 52 (93%) | 0.732 |

**Abbreviations**: AF, atrial fibrillation; BMI, body mass index; EHRA, European Heart Rhythm Association; NOAC, non-vitamin K antagonist oral anticoagulant; TIA; transient ischaemic attack; VKA, vitamin K antagonist

**Supplementary Table S3**. Multivariable regression beta coefficients of the original STOP-Bang items (training cohort).

| **Variable** | **Beta coefficient** |
| --- | --- |
| **Snoring** | 0.073 |
| **Tiredness** | 0.031 |
| **History of observed apnoea** | 0.183 |
| **Hypertension** | 0.184 |
| **BMI >35 kg/m2** | -0.026 |
| **Age >50 years old** | 0.315 |
| **Neck circumference >40cm** | 0.011 |
| **Male** | 0.120 |

**Abbreviations:** BMI, body mass index.

**Supplementary Table S4**. Multivariable regression beta coefficients of AF-specific predictors for moderate-to-severe sleep disordered breathing and corresponding points assigned in the BOSS-GAP score.

| **Variable** | **Beta coefficient** | **Points** |
| --- | --- | --- |
| **Snoring** | 0.048 | 1 |
| **History of observed apnoea** | 0.170 | 2 |
| **Hypertension** | 0.066 | 1 |
| **BMI >=27 kg/m2** | 0.252 | 3 |
| **Age >50 years old** | 0.287 | 3 |
| **Stroke/TIA** | 0.180 | 2 |
| **Male** | 0.104 | 1 |

**Abbreviations:** BMI, body mass index; TIA, transient ischaemic attack.

**Supplementary Table S5: Reclassification table**

| **Moderate to high risk SDB** | | **BOSSGAP >=4** | | **Total** |
| --- | --- | --- | --- | --- |
|  |  | No | Yes |  |
| STOP-Bang >=3 | No | 2 (1.9%) | 15 (14.2%) | 17 (16.0%) |
|  | Yes | 1 (0.9%) | 88 (83.0%) | 89 (84.0%) |
| Total |  | 3 (2.8%) | 103 (97.2%) | 106 (100%) |

| **No to mild risk SDB** | | **BOSSGAP >=4** | | **Total** |
| --- | --- | --- | --- | --- |
|  |  | No | Yes |  |
| STOP-Bang >=3 | No | 21 (21.0%) | 22 (22.0%) | 43 (43.0%) |
|  | Yes | 1 (1.0%) | 56 (56.0%) | 57 (57.0%) |
| Total |  | 22 (22.0%) | 78 (78.0%) | 100 (100%) |

**Abbreviations:** SDB, sleep disordered breathing

**Supplementary Figure S1.** Receiver operating characteristic curve and calibration curves for the performance of the STOP-Bang and the BOSS-GAP scores to predict moderate-to-severe SDB in training (left side) and validation (right side) cohorts.


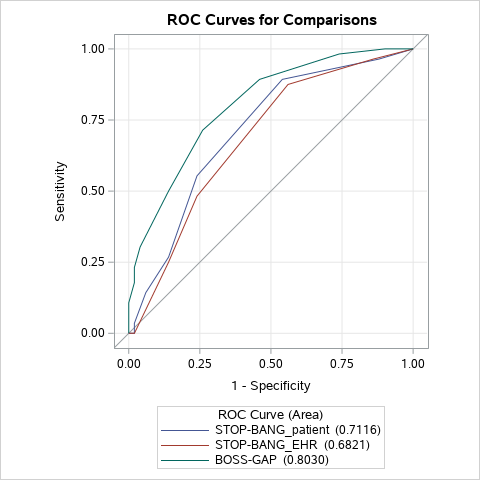

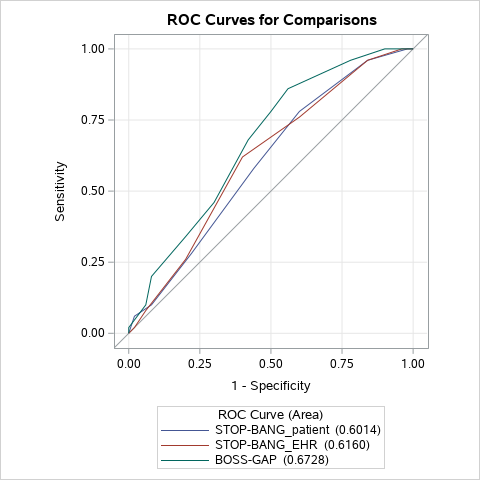


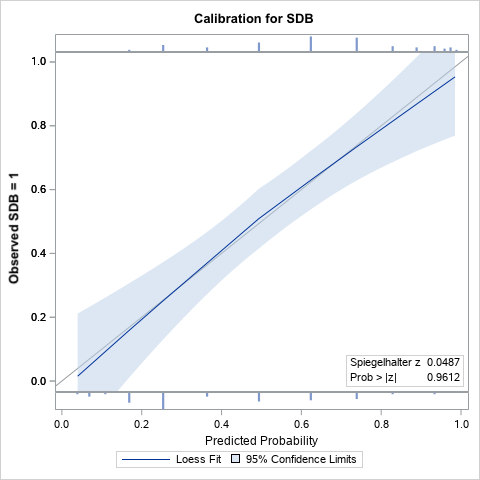

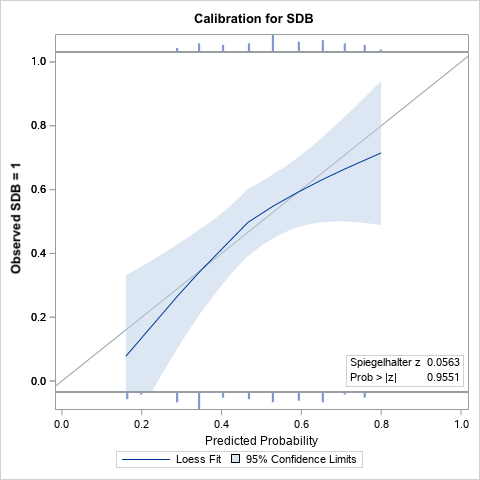


|  | Training cohort; AUROC (95% CI) | Validation cohort;  AUROC (95% CI) |
| --- | --- | --- |
| STOP-Bang (patient-reported items) | 0.712 (0.614-0.810) | 0.601 (0.492-0.711) |
| STOP-Bang (items derived from EHR, where possible) | 0.682 (0.582-0.783) | 0.616 (0.507-0.725) |
| BOSS-GAP | 0.803 (0.721-0.885) | 0.673 (0.568-0.778) |

**Abbreviations:** AUROC, area under the receiver operator characteristic curve; ROC, receiver operator characteristic; SDB, sleep disordered breathing.
